# Role of digital triradius variants and hypothenar patterns in early screening of primary amenorrhea

**DOI:** 10.6026/973206300214616

**Published:** 2025-12-15

**Authors:** Sushil Jiwane, Rekha Jiwane, Vivekanand Gajbhiye

**Affiliations:** 1 Department of Anatomy, Gandhi Medical College, Bhopal, Madhya Pradesh, India; 2 Department of Physiology, AIIMS, Bhopal, Madhya Pradesh, India; 3 Department of Anatomy, RKDF Medical College, Bhopal, Madhya Pradesh, India

**Keywords:** Dermatoglyphics, Actual time of departure (ATD) angle, amenorrhea

## Abstract

Dermatoglyphics, the study of epidermal ridge patterns, is associated with various genetic disorders. Primary amenorrhea, a condition with multifactorial and often genetic etiology, may show distinct dermatoglyphic traits. Hence, the 'ATD' angle was measured in 40 females with primary amenorrhea and 60 healthy controls. Data revealed significantly higher mean 'ATD' angles and greater right-left asymmetry in cases compared to controls. Thus, we show that dermatoglyphic analysis, particularly of the 'ATD' angle, can serve as a simple, non-invasive screening tool for identifying individuals at risk of genetic abnormalities in primary amenorrhea.

## Background:

Dermatoglyphics is a branch of medical science that deals with the study of epidermal ridges and their configuration on the palmar region of the hand and fingers and the plantar region of the foot and toes. The term dermatoglyphics is derived from the Greek words Derma (skin) and Glyphic (to carve) [1]. Dermatoglyphics is one of the recent and advancing branches of Medical Science which depends upon the cornified layer of epidermis and dermal papillae. It is studied and used in the prediction of genetic disorders. Dermatoglyphic patterns form between the 10th and 24th weeks of gestation and remain unchanged throughout life unless the dermis is injured. They are genetically determined and useful in identifying certain congenital disorders [2]. The correlation between dermatoglyphics and other aspects of constitution like disease process is already established [3]. Hence the study of dermatoglyphics proves to be very useful in predicting the hereditary diseases in patients. Abnormal dermatoglyphic patterns are known to occur with genetic disorders like Mongolism, Turner's syndrome, Klinefelter's syndrome etc. Thus, it has been observed that dermatoglyphics shows definite diagnostic changes in disorders with a genetic basis [4]. Moreover, distinctive dermatoglyphic patterns have been documented in several genetic and medical disorders, including Klinefelter syndrome [5] Diagnosis of conditions such as Diabetes Mellitus, Schizophrenia, and Hypertension can now be aided by dermatoglyphic analysis [6]. Studies have shown that individuals with Type 2 Diabetes Mellitus tend to exhibit a higher frequency of loop fingerprint patterns compared to non-diabetic individuals. In contrast, whorl patterns are more commonly observed among non-diabetic participants, whereas arch patterns remain the least frequent in both groups [7]. Since most of the investigations needed to confirm the diagnosis in hereditary disorders are complex and expensive, dermatoglyphics can be efficiently employed with other clinical signs as a screening procedure to define indication for these laboratory procedures. Amenorrhea is one of the major problems encountered by women. WHO Annual Reports 1982 and 1985 have estimated that 15% of the human population is infertile and that amenorrhea is the 6th major cause of female infertility [8]. Amenorrhea, or the absence of menstruation, is a symptom rather than a disease and has a variety of causes, as its occurrence is associated with dysfunction of the hypothalamus, pituitary gland, gonads, or genital ducts [9, 10]. Primary amenorrhea is defined as absence of menstruation by 16 years of age in the presence of normal secondary sexual characters or no menstruation by the age of 14 years of age in the absence of growth of secondary sexual characters [11]. Recent studies from India and other countries have highlighted the ongoing burden of primary amenorrhea and reinforced the strong association with underlying chromosomal and developmental anomalies. Chandel *et al.* (2023) reported diverse cytogenetic patterns in females with primary amenorrhea, underlining the need for early evaluation and genetic counseling [12]. Similarly, Pritti *et al.* (2022) [13] and Nazirudeen *et al.* (2022) documented the etiological spectrum of primary amenorrhea in tertiary care settings, with gonadal dysgenesis and Müllerian anomalies being the leading causes [14]. Rauniyar *et al.* (2024), in a case-control study on females with primary infertility from the Maharashtra population, observed significant differences in dermatoglyphic variables between cases and controls, suggesting their usefulness as a supportive diagnostic or screening tool [15]. Therefore, it is of interest to quantitatively evaluate the 'atd' angle in females with primary amenorrhea and compare it with age-matched healthy controls from the Wardha region, with the aim of identifying a consistent dermatoglyphic marker that may help in screening and early selection of patients for further genetic and cytogenetic evaluation.

## Materials and Methods:

This prospective observational study was carried out after obtaining approval from the Institutional Ethics Committee. Written informed consent was obtained from all participants before enrolment.

## Study population:

A total of 40 diagnosed cases of primary amenorrhea, attending or admitted to the Department of Obstetrics and Gynecology in Central India, were included as the study group. The control group consisted of 60 healthy females with normal menstrual cycles.

## Inclusion criteria (Cases):

Females diagnosed with primary amenorrhea according to WHO criteria: Absence of menstruation by 16 years of age in the presence of normal secondary sexual characters or Absence of menstruation by 14 years of age in the absence of secondary sexual characteristics.

## Exclusion criteria:

[1] Females in the pre-pubertal age group.

[2] Pregnant or lactating women.

[3] Women with known chromosomal syndromes (e.g., Turner's syndrome).

[4] Patients with structural uterine abnormalities.

[5] Post-menopausal women.

[6] Controls were selected only if they had normal clinical examination and did not fall into any exclusion categories.

## Data collection:

A detailed socio-demographic and clinical history was obtained using a semi-structured proforma. Relevant information regarding age, family history, menstrual history, and systemic illness was recorded.

## Dermatoglyphic recording procedure:

Dermatoglyphic prints were obtained using the ink method, which is widely accepted due to its simplicity, cost-effectiveness, and reproducibility.

The method recommended by the American Association of Dermatoglyphics was followed:

[1] The participants were instructed to wash and dry their hands thoroughly to remove dirt and oil.

[2] A thin, uniform layer of duplicating ink was applied on a glass slab.

[3] Each subject was asked to roll their palms and fingers gently over the inked surface, ensuring complete coverage.

[4] Impressions were then carefully transferred onto a clean white paper by pressing the palms and fingers in a controlled manner.

[5] High-quality prints were ensured by repeating the process if smudging or incomplete impressions occurred.

[6] Both right and left hands were recorded for every subject.

## Measurement of 'ATD' Angle:

[1] The 'ATD' angle is formed by joining three triradii points: 'a', 't' and 'd'.

[2] Line 1: from digital triradius 'a' to axial triradius 't'.

[3] Line 2: from axial triradius 't' to digital triradius 'd'.

[4] The angle subtended at point 't' was measured using a protractor.

[5] In cases where more than one axial triradius was present, the widest angle was considered.

[6] Measurements were performed independently by two observers to minimize inter-observer variability.

## Statistical analysis:

The collected data were tabulated and analyzed using descriptive and inferential statistics. Mean and standard deviation (SD) were calculated for ATD angles of both hands. Z-test and Chi-square test were applied to compare mean values and categorical distributions between cases and controls. A p-value of <0.05 was considered statistically significant. Dermatoglyphic prints were obtained using ink method described as per guidelines by American Association of Dermatoglyphics. Dermatoglyphic prints of both hands of each subject were taken, and the 'ATD' angle was calculated. The data was analyzed statistically by using Chi-Square Test.

## 'ATD' angle:

It is the most widely used method in interpreting the position of axial triradius (t). It is formed by lines drawn from digital triradius 'a' to the axial triradius't' and from axial triradius't' to the digital triradius 'd'. In case of more than one axial triradii the widest 'ATD' angle is counted. The more distal the position of t, larger is the 'ATD' angle.

## Results:

The mean ATD angle of the right hand in patients with primary amenorrhea was found to be 44.15° compared to 38.95° in the control group, which was statistically significant. Similarly, the left-hand ATD angle was higher in cases (46.02°) as compared to controls (40.80°). The combined mean (right + left) ATD angle was also significantly higher in patients than in controls, indicating a consistent shift of the axial triradius in the affected group (Table 1 - see PDF). On analysing the distribution of ATD angle ranges, a significantly higher proportion of patients (42.5%) had values greater than 45°, whereas the majority of controls (46.6%) fell in the <40° range, suggesting a strong discriminatory pattern between the two groups (Table 2 - see PDF). Right-left asymmetry of the ATD angle (absolute difference >2°) was observed in 52.5% of cases as compared to only 18.3% of controls, indicating greater developmental instability in patients with primary amenorrhea (Table 3 - see PDF).

## Discussion:

The present study demonstrates that the mean ATD angle is significantly higher in patients with primary amenorrhea as compared to the control group, both in the right and left hands. These findings suggest that dermatoglyphic traits, particularly the atd angle, may serve as a useful non-invasive marker for identifying individuals at risk of underlying genetic abnormalities associated with primary amenorrhea. The increased ATD angle likely arises due to distal displacement of the axial triradius, which reflects disturbances in embryological development. Since dermatoglyphic patterns are formed during 12-24 weeks of intrauterine life and remain unchanged thereafter, they provide a permanent record of intrauterine growth processes [12]. Abnormal dermatoglyphic features have long been associated with genetic syndromes, such as Turner's, Klinefelter's and Down syndrome [5]. Since primary amenorrhea often has a genetic or chromosomal etiology, the increased ATD angle observed in this study may be a reflection of similar underlying mechanisms. In fact, studies such as those by Joseph & Thomas (1982) [13] have reported chromosomal abnormalities in a large proportion of primary amenorrhea cases. Thus, dermatoglyphic screening could serve as a cost-effective preliminary tool before cytogenetic or hormonal assays, particularly in resource-constrained settings. Despite these encouraging findings; limitations of the present study should be acknowledged. The sample size was relatively small and confined to a single geographic region, which may affect generalizability. Additionally, the ink method, though inexpensive and widely used, is prone to variability in print quality, which could affect measurement accuracy. Future research with larger, multi-centric cohorts and integration of dermatoglyphic findings with cytogenetic analysis would provide stronger evidence and clinical applicability. Nevertheless, our study emphasizes that dermatoglyphics, being simple, non-invasive, and economical, can act as an important screening tool in the evaluation of primary amenorrhea. Since amenorrhea is a significant contributor to infertility in young women [8], early detection through dermatoglyphic traits such as ATD angle may aid in timely diagnosis and management. ATD angle shows statistical significance in right, left and both (right and left hand) in present study i.e. ATD angle is increased in primary amenorrhea patients as compared to controls which are an important parameter. Recent research continues to support the association between dermatoglyphic traits (including ATD angles) and conditions with genetic or developmental bases. A study done by Rauniyar *et al.* [15] in a 2024 case-control study in Maharashtra comparing females with primary infertility versus healthy controls found that infertile females had a significantly higher mean ATD angle and lower total ridge counts. This suggests that not only amenorrhea, but other disorders of reproductive function show similar dermatoglyphic deviations, possibly reflecting early embryonic development disturbances. Also, though in a different clinical domain, recent work among Type 2 Diabetes Mellitus patients done by Shrestha *et al.* [16] has re-affirmed that mean ATD angle is elevated relative to normative reference values. Although diabetes is not directly related to amenorrhea, this consistency in findings across pathologies with genetic/environmental influences supports the idea that alterations in ATD angle reflect early developmental perturbations. In relation to our findings, if we observe a significantly higher ATD angle in primary amenorrhea cases compared to controls, this is in line with what has been observed in infertility research and in other conditions involving developmental or chromosomal disturbances. It may support the hypothesis that the genetic or embryonic factors influencing ovarian development or hormonal axis formation also influence palm ridge formation (axial triradius position).

## Conclusion:

A statistically significant increase in the mean ATD angle and right-left asymmetry among patients with primary amenorrhea compared to controls. Data shows that dermatoglyphic traits, particularly the ATD angle, can serve as simple, non-invasive markers of underlying genetic or developmental disturbances. Thus, dermatoglyphic analysis may be a useful screening tool for early identification and referral of primary amenorrhea cases, especially in resource-limited settings.
